# Chocolate Bushbabies: Cocoa Agroforests as Habitat for Galagos (Galagidae) in the Ashanti Region, Ghana

**DOI:** 10.1002/ece3.74026

**Published:** 2026-07-15

**Authors:** Daniel Hending, Joseph Anokye, Lubasi Limweta, Mohammed Abdul Razak Yombu, Florence Quainoo‐Mensah, David Gorleku, Thomas M. Butynski, Yvonne A. de Jong, Magdalena S. Svensson, Phoebe Reid, Ricardo Rocha, Evans Dawoe, William Thompson

**Affiliations:** ^1^ Department of Biology University of Oxford Oxford UK; ^2^ Department of Agroforestry, Faculty of Renewable Natural Resources Kwame Nkrumah University of Science and Technology Kumasi Ghana; ^3^ Eastern Africa Primate Diversity and Conservation Program Nanyuki Kenya; ^4^ Nocturnal Primate Research Group Oxford Brookes University Oxford UK

**Keywords:** acoustic survey, automatic classifier, forest matrix, habitat use, primates, Strepsirrhini

## Abstract

Increased demand for arable land and pastures is a key driver of deforestation in the tropics. Arboreal primates are particularly threatened by deforestation, and although many primate species are restricted to undisturbed natural forests, some can persist in anthropogenic habitats such as plantations and agroforests. In this study, we conducted passive acoustic surveys for galagos (Family: Galagidae) in 38 cocoa agroforests in the Ashanti Region of Ghana. We trained a YOLOv5 convolutional neural network model using a dataset of galago vocalisations from across sub‐Saharan Africa to enable automatic detection of galago calls. Despite a high rate of false‐positive detections (95%), our galago detector model confirmed the presence of at least one species of western dwarf galago (*Galagoides*) in four cocoa agroforests, each situated within 500 m of a remnant forest patch, but > 4 km from the nearest forest reserve. These represent the first scientific records of galagos in cocoa agroforestry systems, although local ecological knowledge likely predates these observations. Further work is needed to determine whether cocoa agroforests form viable habitats for galagos, or whether they function as travel vectors between remnant forest fragments, something that our galago detector can facilitate via passive acoustic monitoring.

## Graphical abstract

We assessed whether galagos occur in cocoa agroforestry systems in the Ashanti Region of Ghana, using passive acoustic monitoring and a YOLOv5‐based convolutional neural network trained on galago vocalisations from sub‐Saharan Africa. We confirmed the presence of at least one species of western dwarf galago (Galagoides sp.) in four cocoa agroforests located more than 4 km from the nearest forest reserve, and these findings are the first scientific evidence of galagos inhabiting cocoa agroforestry systems. This study demonstrates the applicability of machine learning‐assisted acoustic monitoring for detecting cryptic species and provides a foundation for future work evaluating whether agroforests serve as viable habitats or movement corridors for galagos in fragmented tropical ecosystems.

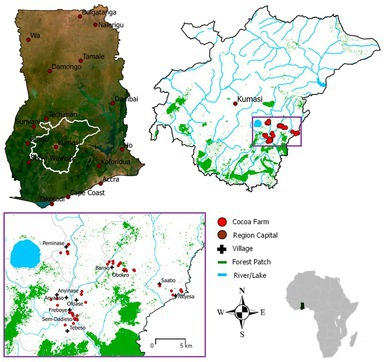

## 1 Introduction

Agricultural expansion for croplands, plantations and pastures, driven by human population growth, is a leading cause of deforestation in the tropics (Foley et al. 2005; Maja and Ayano 2021; Pendrill et al. 2022). Tropical forests are vital habitats for around half of the world's vertebrate species (Pillay et al. 2022), and deforestation and expanding agriculture are therefore major drivers of biodiversity loss and species extinction (Williams et al. 2021; Wiebe and Wilcove 2025). Understanding how species respond to land‐cover changes is therefore important for protecting tropical forests and ensuring the long‐term survival of species (Kinnaird et al. 2003; Gonthier et al. 2014).

Primates are particularly threatened by agricultural expansion and deforestation, as approximately 90% of primate species are dependent on forest cover for survival (Chapman et al. 2006; Isabirye‐Basuta and Lwanga 2008). Further, more than 60% of primates are currently listed as Vulnerable, Endangered or Critically Endangered on the IUCN Red List of Threatened Species (Fernández et al. 2022; Wallis 2023; IUCN 2026). Many primates are sensitive to changes in their habitat, food availability and anthropogenic activity (e.g., Lambert and Rothman 2015; de Almeida‐Rocha et al. 2017; Hending et al. 2024), and forest degradation, loss and fragmentation therefore represent major drivers of population declines and extirpations (Estrada et al. 2017). However, some ecological generalist primates demonstrate resilience to habitat degradation, and they may inhabit sub‐optimal anthropogenic habitats (Gumert et al. 2011; Knoop et al. 2017; Hending 2021).

Agroforests are among these anthropogenic habitats (Estrada et al. 2012; Butynski and de Jong 2014). For example, primates occur in plantations of cocoa *Theobroma cacao* (Webber et al. 2019), vanilla *Vanilla planifolia* (Hending et al. 2018a), cashew *Anacardium occidentale* (Hockings and Sousa 2012), coffee *Coffea* spp. (Merker and Muhlenberg 2000) and oil palm *Elaeis* spp. (Campbell‐Smith et al. 2010). Due to their adaptability to secondary vegetation, strepsirrhines are common primates in some agricultural landscapes (e.g., Hending et al. 2018b), as well as larger‐bodied primates such as chimpanzee *Pan troglodytes* (McLennan et al. 2021), baboons *Papio* spp., savanna monkeys *Chlorocebus* spp., spider monkeys *Ateles* spp. (Arroyo‐Rodríguez et al. 2017) and lemurs (Ganzhorn 1987).

Galagos (Family: Galagidae), or bushbabies, are small, nocturnal, strepsirrhines endemic to sub‐Saharan Africa (Groves 2005). The galagids comprise 20 species in six genera (Butynski et al. 2013; Penna and Pozzi 2024), all of which require trees for travel, food and resting (Butynski et al. 2006; Svensson et al. 2018; Ellison et al. 2019). Galagos spend the day resting in tree hollows, dense vegetation, or nests (Ellison et al. 2019) and although they often forage alone, social organisation is complex, with individuals often sharing sleeping sites and maintaining contact through vocalisations, scent marking and overlapping home ranges (Ellison et al. 2024). Home ranges are small (0.5–3.0 Ha; Harcourt and Nash 1986; Ambrose and Butynski 2013a, 2013b; Nowack et al. 2013), but individual galagos may travel up to 2 km per night within their range and dispersal distances of several kilometres have been observed (Nash and Harcourt 1986). Galago diets are diverse and encompass insects, fruits and tree gum (Harcourt 1986). All galagos are, to some extent, threatened by forest, woodland and bushland degradation, loss and fragmentation (Rosti et al. 2024). This degradation, loss and fragmentation is extensive and occurring at a high rate over much of sub‐Saharan Africa (Rudel 2013; Taubert et al. 2018) due to logging and conversion to croplands and livestock grazing lands (Ordway et al. 2017; Jellason et al. 2021).

Galagos are among the least‐studied of primates (Penna and Pozzi 2024) and further information on habitat use and adaptations to agriculture is both of scientific interest and essential to their long‐term survival (Estrada et al. 2012). The geographic ranges of the galagids are predominantly characterised by a matrix of agricultural land, forest corridors and remnant forest patches (Aleman et al. 2018). Galagos exhibit some adaptability and resilience to habitat degradation and land cover change (Nash et al. 1989; Scheun et al. 2015; Forbanka 2018; Scheun and Nowack 2024), surviving in cashew plantations (Svensson and Bearder 2013), clove and coconut plantations (Masters et al. 1988), firewood plantations (Estrada et al. 2012), mixed tree plantations (Happold and Happold 2013) and rangelands (Butynski and de Jong 2014), but their use of many other agroecosystems remains unknown. Galagos are not reported as crop pests when occurring in agroecosystems (Estrada et al. 2012).

Cocoa is the most important cash crop in West Africa, with two million households dependent on cocoa cultivation as their primary source of income (Ingram et al. 2018). Approximately 70% of the world's cocoa is grown in West Africa (Schroth et al. 2016) and large areas of Cote d'Ivoire, Ghana, Nigeria and Cameroon are now characterised by matrices of cocoa agroforests and remnant forest patches (Becker et al. 2025; Koralewicz et al. 2025). Cocoa in West Africa is commonly grown alongside other crops and native trees (Koko et al. 2013; Asare et al. 2019). In this study, we assessed the presence of galagos in a cocoa cultivation landscape in the Ashanti Region of Ghana. As agroforests provide travel routes, food and shelter for many species, potentially including galagos (Guzmán et al. 2016; Bersacola et al. 2022; Hending et al. 2023) and as three species of galago are known to occur throughout West Africa, the Senegal Galago ( *Galago senegalensis* ), Demidoff's Dwarf Galago ( *Galagoides demidoff* ) and Thomas's Dwarf Galago ( *Galagoides thomasi* ) (Penna and Pozzi 2024), we predicted that we would detect galagos in Ghana's cocoa farms.

## 2 Methods

### 2.1 Study Area

**FIGURE 1:**
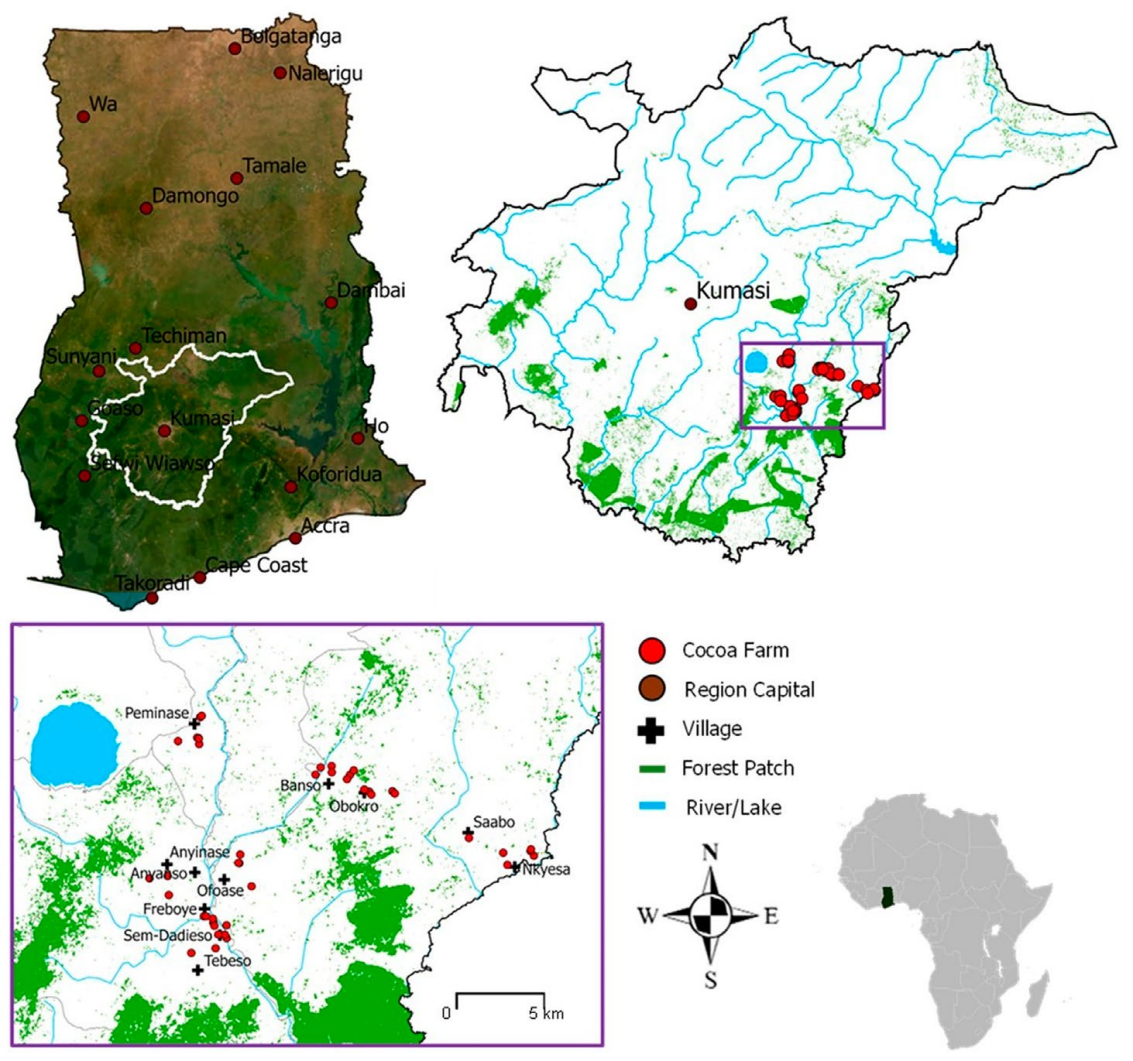
Ashanti Region, Ghana and the location of the 38 study farms within Asante Akim Central, Asante Akim South and Bosome Freho Districts. Green represents remaining natural forest. Maps created using ArcGIS Pro, with a scale of 1:5,000,000 for Ghana, 1:1200,00 for the Ashanti Region and 1:100,000 for the zoomed map.

This study was conducted in Asante Akim Central, Asante Akim South and Bosome Freho Districts of the Ashanti Region, Ghana (hereafter referred to as ‘Ashanti’, Figure 1; Asamoah et al. 2017). Ashanti Region (24,389 km^2^; Asamoah et al. 2017) is situated within Ghana's primary cocoa‐cultivation zone and is characterised by a matrix of cocoa farms, patches of dry and moist semi‐deciduous forest and illegal gold mines (Nti et al. 2020). Eighty‐five percent of Ghana's cocoa is produced in the Ashanti Region, and the neighbouring Western and Brong Ahafo Regions (Asante‐Poku and Angelucci 2013). Ashanti Region falls within the wet semi‐equatorial climatic zone with a mean annual rainfall of 170–185 cm, most of which falls in the March–July and September–November periods (Asamoah et al. 2017). Mean annual temperatures are 25°C–32°C, with an annual humidity range of 65%–85% (Dwamena et al. 2022). Ashanti lies within the Guinean Forests of West Africa global biodiversity hotspot (Myers et al. 2000), and the region harbours approximately 25 separate forest reserves and several threatened species, including pangolins and White‐naped Mangabeys (Wiafe 2021; Sampson and Appiah‐Opoku 2026). This study was conducted in 38 cocoa farms around 11 communities (Figure 1). All farms were shade tree cocoa farms (mean shade cover = 15%; mean shade tree height = 12.1 m), located within a large expanse of cocoa agroforests that are intersected by other agricultural plots including corn and rice fields.

### 2.2 Passive Acoustic Surveys

Like many other nocturnal Strepsirrhines, galagos are highly vocal as they need to coordinate spacing and advertise reproductive state (Seiler et al. 2019; Schneiderová et al. 2020). Further, they also have a species‐specific vocal repertoire, including a range of loud calls such as the crescendo, shriek, trill, whistle and yap (Zimmermann 1990; Butynski et al. 2006; Bearder et al. 2013; Bettridge et al. 2019). These elusive, small‐bodied primates are, therefore, often easier to detect acoustically than visually (Nekaris et al. 2008; Hending et al. 2020). To detect galagos, we conducted passive acoustic surveys within the 38 cocoa farms during September—November 2024 (mean size = 3.06 ha, range = 1.13–6.55 ha). We deployed one Audiomoth (Hill et al. 2018) in the centre of each cocoa farm and set it to record for the period 18:00–05:00 at a 384 kHz sampling rate with mid‐gain settings (sampling rate was high as bat activity was surveyed in parallel). With these settings, the Audiomoths would be able to detect galago vocalisations from approximately 50–100 m away (Entling et al. 2026), although signal attenuation and therefore detection distance, would vary greatly between sites with different vegetation characteristics (Maciej et al. 2011). Following Araújo‐Fernandes et al. (2025), we programmed Audiomoths to record for only 1 min every 5 min to save memory storage space and enhance battery life. Total recording time per day within this 11‐h window was, therefore, 132 min. We surveyed each farm for approximately 14 days. Audiomoths were stored in standard Audiomoth cases and attached to a shade tree at about 2 m above ground. All acoustic data were stored internally on 128 GB micro‐SD cards.

### 2.3 Galago Detector

To locate and isolate galago vocalisations among the passive acoustic recordings, we used the Ultralytics YOLOv5 Computer Vision Model (Jocher et al. 2022). YOLOv5 is a convolutional neural network (CNN) that can be applied for machine learning‐based recognition of specific image patterns in spectrograms (Husain and Osawa 2023) and has been applied to find signals of interest in ecology and conservation studies (e.g., Escobar‐Amado et al. 2023; Vo et al. 2023). We first compiled a collection of reference calls of the three galago species known to occur in Ghana ( *Galago senegalensis* , *Galagoides demidoff* , *Galagoides thomasi* ) (Oates 2011; Ambrose and Butynski 2013a, 2013b; Nash et al. 2013; Penna and Pozzi 2024). To train the YOLOv5 model, we then manually annotated all reference vocalisations in Raven Lite using bounding boxes. Each annotation included the minimum and maximum frequency of the call, the start and end time of the call within its respective audio file and an annotation tag identifying species and call type. Given that galagos have a repertoire of context‐specific calls, we assigned the call type annotation to each call based on published vocal repertoires (Zimmermann 1985; Masters 1991; Schneiderová et al. 2016). A total of 1915 calls were annotated to serve as the training dataset for the CNN. The number of reference calls for each call type and each species was highly variable (Supplementary File S1).

The YOLOv5 CNN was trained using the dataset in Python over 100 epochs and a batch size of 16. The total training data set was split into three subsets for model training (60%), testing (20%) and validation (20%). Following standard machine learning approaches, we included the validation data to evaluate model performance. The test data were withheld for computation of performance metrics only (McCammon et al. 2025). The trained model evaluation metrics suggested moderate precision performance (0.390) and recall (0.580), and good validation box loss (0.100), but uneven classification accuracy among call types. This was almost certainly due to (1) high variability in the number of each call type, and (2) the low number of reference calls for some call types (e.g., *G. senegalensis* whistle, File S1). Since the purpose of this study was to detect galagos rather than discriminate among species and call types, we proceeded to apply our model to the acoustic data. We used a high‐performance computer with an RTX8000 GPU, 8 CPUs and 32 GB of RAM assigned to each CPU to scan the acoustic data for galago calls. The trained model was programmed to loop through all files, scanning each 1‐min‐long wav file in 10 s chunks (spectrograms). A confidence threshold of 0.500 was applied. All results were saved as csv files. Finally, we manually checked all recorded detection instances and recorded the sites where galagos were confirmed to be present.

### 2.4 Spatial Analysis

To determine how far outside of forest patches galagos occur, we generated a forest spatial layer for Ashanti Region. We used the global canopy height layer (10 m^2^ resolution; Lang et al. 2023) and clipped this to include only pixels with an average height of ≥ 20 m. This enabled us to differentiate between forest and cocoa, which only grows to 8 m and agroforest shade trees, which may grow tall but are in lower density than natural forest; mean shade tree height on our farms was 12.1 m, so a ≥ 20 m cutoff threshold for the canopy height layer also enabled us to differentiate natural forest from agroforest (i.e., shade trees on cocoa farms). Using ArcGIS Pro's spatial analysis tools, we calculated the minimum distance from the boundary of each cocoa farm to the nearest forest layer pixel (forest patch). In addition, using a shapefile of Ghana's forest reserves, we calculated the minimum distance from the boundary of each cocoa farm to the boundary of the nearest forest reserve (protected area).

## 3 Results

A total of 1605 potential galago vocalisations were detected by our detector within the acoustic survey dataset: 1299 yaps, 241 crescendos, 65 honks and 1 trill. All yaps and honks detected were false positives, where the model incorrectly classified signals produced by insects. A large proportion of the crescendo calls were also false positives, as they resembled insect chitters. Crescendo calls at four farms were correctly detected by the model and manually confirmed via inspection of the acoustic files and their accompanying spectrograms. In total, galago vocalisations were detected at four of the 38 cocoa farms (10.5%); however, the species identity of the callers could not be identified by our detector.

The 38 farms were located a mean distance of 325 m from the nearest forest patch (minimum = 20 m, maximum = 867 m). The four farms in which we detected galagos were located within proximity of three communities: Anyanso (*N* = 1), Banso (*N* = 1) and Nkyesa (*N* = 2) (Table 1). These farms were 35 m (Nkyesa 1), 133 m (Nkyesa 3), 139 m (Banso 2) and 455 m (Anyanso 1) from the nearest forest patch. As the forest layer used was a clipped version of the 10 m^2^ global canopy height layer, forest patches may merely represent 10 × 10 m areas of tall trees (Figure 1). These farms were about 4426 m (Anyanso 1), 17,997 m (Banso 2), 28,345 m (Nkyesa 1) and 28,277 m (Nkyesa 3) from the nearest intact forest reserve (Table 1). Galagos were detected on farms located at a greater than average distance (1461 m) away from the nearest community (mean = 2156 m, range = 1672–2825 m). The farms where galagos were detected were also situated in closer proximity to the nearest forest patch (mean = 190 m, range = 35–455 m) than the average value for all farms (327 m); however, the nearest forest patches to farms with galagos were smaller (mean = 1.162 ha, range = 0.017–3.785 ha) than the mean nearest patch size (1.937 ha).

**TABLE 1:** Summary of the 38 study farms in the Ashanti Region, Ghana, their total area size, their distance from their respective communities, their distance from the nearest forest patch and nearest forest reserve and the total area size of the nearest forest patch and the nearest forest reserve.

Farm name	Farm area (ha)	Community	Galagos detected	Distance to community (m)	Distance to forest patch (m)	Forest patch size (ha)	Distance to forest reserve (m)	Forest reserve size (ha)
Anyanso 1	0.947	Anyanso	Yes	2825	455	0.801	4426	7728
Anyinase 1	0.314	Anyinase	No	2060	192	0.460	2107	7728
Anyinase 2	0.599	Anyinase	No	1069	382	0.051	3029	7728
Banso 1	1.351	Banso	No	2115	210	0.724	20,239	7728
Banso 2	0.196	Banso	Yes	1672	139	0.017	17,997	7728
Banso 3	0.611	Banso	No	1651	42	0.051	18,972	7728
Banso 4	0.506	Banso	No	1754	85	0.034	19,843	7728
Banso 5	0.721	Banso	No	2286	247	49.549	20,690	7728
Banso 6	1.183	Banso	No	1102	20	0.026	18,756	7728
Freboye 1	0.627	Freboye	No	693	800	0.290	7397	7252
Freboye 2	1.351	Freboye	No	1297	168	0.145	6958	7252
Freboye 3	0.500	Freboye	No	990	343	0.145	6712	7252
Freboye 4	1.027	Freboye	No	1082	515	7.120	6792	7252
Freboye 5	1.125	Freboye	No	1177	225	0.051	7327	7252
Freboye 6	0.652	Freboye	No	704	867	0.051	7426	7252
Nkyesa 1	0.824	Nkyesa	Yes	2052	35	0.043	28,345	7252
Nkyesa 2	1.306	Nkyesa	No	1684	148	0.333	25,987	7252
Nkyesa 3	1.448	Nkyesa	Yes	2073	133	3.785	28,277	7252
Nkyesa 4	0.812	Nkyesa	No	701	100	0.034	25,781	7252
Obokro 1	0.416	Obokro	No	2597	248	0.034	22,492	7252
Obokro 2	1.512	Obokro	No	471	390	0.119	21,387	7252
Obokro 3	0.762	Obokro	No	662	95	0.119	21,242	7252
Obokro 4	0.790	Obokro	No	305	479	0.520	21,055	7728
Obokro 5	2.184	Obokro	No	2817	146	0.034	22,440	7252
Ofoase 1	5.343	Ofoase	No	2014	184	2.822	8764	7728
Ofoase 2	1.487	Ofoase	No	2733	175	0.102	8813	7728
Ofoase 3	1.195	Ofoase	No	2585	99	0.469	10,082	7252
Peminase 1	0.597	Peminase	No	1410	804	0.179	11,018	7728
Peminase 2	0.360	Peminase	No	2193	58	0.068	9938	7728
Peminase 3	0.618	Peminase	No	1291	837	0.034	10,995	7728
Peminase 4	4.222	Peminase	No	1911	321	0.085	10,570	7728
Peminase 5	1.743	Peminase	No	806	506	0.017	12,787	7728
Saabo 1	0.312	Saabo	No	484	104	0.128	24,136	7252
Sem‐Dadieso 1	0.228	Sem‐Dadieso	No	663	637	2.285	5679	7252
Sem‐Dadieso 2	0.738	Sem‐Dadieso	No	454	692	2.285	6045	7252
Sem‐Dadieso 3	0.757	Sem‐Dadieso	No	168	569	0.068	6078	7252
Tebeso 1	4.399	Tebeso	No	1702	372	0.060	3992	7252
Tebeso 2	1.031	Tebeso	No	1266	519	0.469	4809	7728

## 4 Discussion

### 4.1 Galago Detector Performance

Although the galago detector was able to identify 1605 potential galago calls within our data set, 95% of these were false positives. Upon manual verification of the detection instances, it was apparent that some call types, such as the yap and trill, occupy the same frequency bands as insect signals and, therefore, appear spectrographically similar. These frequency overlaps among galago vocalisations and insect signals have been reported in other galago species (Génin 2021). While the performance of the model was poor due to the high percentage of false positive detections, it did detect galagos in four cocoa agroforests as per our prediction. The evaluation metrics of the detector suggest that it could identify galago calls if present within an audio file. Missed detections cannot, however, be ruled out (Teixeira et al. 2019) and galagos may be present in some or all of the other 34 cocoa agroforests.

The use of bioacoustics is increasingly common in biodiversity surveys (e.g., Sueur et al. 2008; Darras et al. 2025; Hending et al. 2025). Call classifiers such as the Ultralytics YOLOv5 computer vision model are highly useful for automatically isolating signals from acoustic data without having to manually scan through hundreds of hours of audio recordings (Emmett et al. 2020; Wood et al. 2023; Batist et al. 2024). Our galago detector's performance could be improved by expanding the training dataset to contain more calls of each type (Shahinfar et al. 2020) and by ensuring a more even distribution of training data among call types (Miao et al. 2019). This will enable further surveys in other habitats of the three species of galago on which our model was trained. Further, additional automated detection tools such as monitoR (Katz et al. 2016) or Arbimon (Aide et al. 2013) were not utilised in this study, and they could be tested in future to improve model performance. Additionally, if training data for additional species are available, the detector could be expanded to cover more taxa on a broader taxonomic and geographic scale. This would greatly facilitate the passive field identification of galagos, which are notoriously difficult to identify in situ (Pozzi et al. 2019; Ellison et al. 2021).

### 4.2 Species Identity

The only confirmed call types detected by this study were crescendo calls. Based on what is known about the vocal profiles (Wild Solutions 2026) and geographic ranges of the three galago species in this study, the crescendo calls recorded are those of either *G. demidoff* or *G. thomasi* (Ambrose and Butynski 2013a, 2013b; Penna and Pozzi 2024). Due to the uneven number of calls among each call type of each species for the training data, the detector was unable to determine which of the two species the detected calls belonged to (File S1). Despite this, we can therefore state that *Galagoides* sp. occurs within the cocoa farms in our study area. Of the two *Galagoides* species, *G. demidoff* is the most likely identity of the detections, as this species tends to occupy low, secondary growth habitat, including logged and degraded forests, even sometimes moving into tall grassland (Ambrose 1999; Ambrose and Butynski 2013a; Bersacola et al. 2015). Although *G. thomasi* occurs in plantations and farmlands, this species has a preference for primary and secondary natural forests (Ambrose and Butynski 2013b) and is therefore less likely than *G. demidoff* to occupy cocoa agroforests. The species identity of our detected galagos warrants further study.

### 4.3 Conservation Implications and Further Research

We detected Galagos on only four of the 38 cocoa farms surveyed, representing the first records of galagos in cocoa agroforests in Ghana. Although the small number of detections limits our inference of these findings, all of the occupied farms were located within 500 m of a forest patch, and three of the four occupied farms were within 150 m of the nearest patch (Table 1). This pattern suggests that galago use of cocoa agroforests is facilitated by proximity to remnant forest habitat (Estrada et al. 2012). Farm occupancy was recorded not only near relatively large forest fragments, but also on one farm adjacent to an extremely small forest patch of only 0.017 ha (Table 1). This suggests that galagos may be able to use very small patches of remnant forest as stepping stone habitats within landscapes characterised predominantly by agricultural land. While we did not assess the quality and structure of these forest patch habitats, this observation suggests that very small forest remnants may function as stepping stones for galago dispersal through agricultural landscapes (Anderson et al. 2007). The occurrence of galagos on farms located many kilometres from the nearest forest reserve further indicates that local habitat remnants may be more relevant to agroforest occupancy than distance to large protected forests. Given the generally small size of the surveyed farms and the low overall galago detection rate, individual cocoa farms are unlikely to support independent populations. Instead, galago occurrence within cocoa agroforests may reflect a system where individuals are dispersing and recolonising habitat patches, with remnant forests acting as source habitats and movement corridors within the predominantly agricultural landscape (Anderson et al. 2007; Nasi et al. 2008). Under this scenario, the absence of galagos from many farms may result from local extinction in isolated patches or from limited dispersal opportunities (Benchimol and Peres 2014), highlighting the potential conservation value of retaining even small forest remnants within cocoa‐dominated landscapes (Beard et al. 2025). Further work into galago occupancy of agroforests is needed, and this should utilise a combination of methods including line transects and camera traps, in order to cross‐validate our preliminary results from our passive acoustic surveys.

Ashanti Region, and indeed much of southern Ghana, is characterised by large areas of cocoa plantations (Asante‐Poku and Angelucci 2013; Nti et al. 2020). With the exception of the small forest patches within the study area (Figure 1), all surveyed farms were located within a large expanse of cocoa agroforest intersected by other agricultural plots. *Galagoides* home ranges are small (0.5–3.0 Ha; Harcourt and Nash 1986; Ambrose and Butynski 2013a, 2013b; Nowack et al. 2013), and so they would be unable to travel large distances., suggesting that individuals detected in cocoa farms must spend at least part of their lives within these agricultural landscapes rather than relying exclusively on larger forest reserves. However, as we were unable to conduct acoustic surveys within the surrounding forest patches themselves as part of this investigation, we cannot determine whether galagos were resident within cocoa farms or whether they were periodically moving into agricultural areas from adjacent forest to forage. Despite this, our detections indicate that cocoa agroforests are used by *Galagoides* sp. and may provide suitable habitat, as has been observed in other plantation systems for multiple primate species (Butynski and de Jong 2014; Gérard et al. 2015; Guzmán et al. 2016; Hending et al. 2018a; Webber et al. 2019; Franquesa‐Soler et al. 2022). These findings are, therefore, encouraging for galago conservation.

The presence of at least one species of *Galagoides* in cocoa farms may be beneficial to farmers as they contribute to seed dispersal which can improve soil health (Abondano et al. 2023; Ganzhorn et al. 2024; Ramananjato 2025), and their insectivorous diets may suppress pests. However, future research is needed to determine whether remnant forest patches within Ghana's cocoa‐growing regions are stepping stones or source habitats for galagos, and whether cocoa agroforests are used as travel routes through which galagos move to access neighbouring forest patches (Bersacola et al. 2022). With additional training data, our detector model has the potential to answer this research question for multiple galago taxa across sub‐Saharan Africa. Further, the impact of galagos on farms (albeit whether they are permanent residents or temporary occupants) should be assessed to determine whether they are potentially crop pests or drivers of ecosystem services, something that is not currently well understood (Hill 2005). Galagos are threatened by habitat loss, climate change and the illegal pet trade (Svensson et al. 2021; Scheun and Nowack 2024). Our findings showcase the flexibility of galagos to persist within landscapes dominated by agroecosystems, and are therefore encouraging for their conservation.

## Author Contributions

**Daniel Hending:** conceptualization (lead), data curation (lead), formal analysis (lead), investigation (equal), methodology (lead), project administration (equal), resources (lead), software (lead), supervision (equal), validation (lead), visualization (lead), writing – original draft (lead), writing – review and editing (equal). **Ricardo Rocha:** resources (supporting), supervision (supporting), writing – review and editing (equal). **Phoebe Reid:** investigation (equal), writing – review and editing (equal). **Magdalena S. Svensson:** resources (supporting), validation (supporting), writing – review and editing (equal). **Evans Dawoe:** resources (supporting), supervision (equal), writing – review and editing (equal). **William Thompson:** conceptualization (supporting), funding acquisition (lead), supervision (equal), writing – review and editing (equal). **Mohammed Abdul Razak Yombu:** investigation (equal), writing – review and editing (equal). **Lubasi Limweta:** investigation (equal), writing – review and editing (equal). **Joseph Anokye:** investigation (equal), writing – review and editing (equal). **Florence Quainoo‐Mensah:** investigation (equal), writing – review and editing (equal). **Yvonne A. de Jong:** resources (supporting), validation (supporting), writing – review and editing (equal). **Thomas M. Butynski:** resources (supporting), validation (supporting), writing – review and editing (equal). **David Gorleku:** investigation (equal), writing – review and editing (equal).

## Funding

This work was supported by Global Centre on Biodiversity for Climate, G01‐007652.

## Conflicts of Interest

The authors declare no conflicts of interest.

## Supporting information


**File S1:** Number of reference call annotations of each call type of three species of galago used to train a YOLOv5 galago detecting model for a passive acoustic survey of 38 cocoa farms in the Ashanti Region, Ghana.

## Data Availability

The associated data for this work is available on Dryad: https://doi.org/10.5061/dryad.kwh70rzm0.
