# Supplementary material for: Chocolate Bushbabies: Cocoa Agroforests as Habitat for Galagos (Galagidae) in the Ashanti Region, Ghana
Source: Ecol Evol. 2026 Jul 15;16(7):e74026. doi: 10.1002/ece3.74026 (PMC13373318; doi:10.1002/ece3.74026)
Supplement: Supplementary file 1 — File S1: Number of reference call annotations of each call type of three species of galago used to train a YOLOv5 galago detecting model for a passive acoustic survey of 38 cocoa farms in the Ashanti Region, Ghana. [file ECE3-16-e74026-s001.docx]

**Supplementary File 1:** Number of reference call annotations of each call type of three species of galago used to train a YOLOv5 galago detecting model for a passive acoustic survey of 38 cocoa farms in the Ashanti Region, Ghana.

| **Call Type** | **Species** | | |
| --- | --- | --- | --- |
|  | ***Galago senegalensis*** | ***Galagoides demidoff*** | ***Galagoides thomasi*** |
| **Advert** | 34 | 0 | 0 |
| **Alarm** | 121 | 0 | 0 |
| **Buzz** | 78 | 83 | 0 |
| **Chatter** | 0 | 44 | 0 |
| **Crescendo** | 0 | 83 | 85 |
| **Grunt** | 0 | 90 | 0 |
| **Honk** | 241 | 0 | 0 |
| **Shriek** | 0 | 0 | 31 |
| **Tjong** | 92 | 0 | 0 |
| **Trill** | 0 | 0 | 21 |
| **Whistle** | 9 | 0 | 0 |
| **Yap** | 256 | 478 | 169 |
